# The impact of egg incubation temperature on the personality of oviparous reptiles

**DOI:** 10.1007/s10071-016-1030-1

**Published:** 2016-09-06

**Authors:** Harry Siviter, D. Charles Deeming, Joanna Rosenberger, Oliver H. P. Burman, Sophie A. Moszuti, Anna Wilkinson

**Affiliations:** 1 School of Life Sciences, University of Lincoln, Lincoln, LN6 7DL UK; 2 Institute of Animal Breeding, Division of Poultry Breeding, Wroclaw University of Environmental and Life Sciences, Wrocław, Poland

**Keywords:** Personality, Incubation temperature, Boldness, Behavioral trait, Lizard, Reptile

## Abstract

Personality traits, defined as differences in the behavior of individual animals of the same species that are consistent over time and context, such as ‘boldness,’ have been shown to be both heritable and be influenced by external factors, such as predation pressure. Currently, we know very little about the role that early environmental factors have upon personality. Thus, we investigated the impact of incubation temperature upon the boldness on an oviparous reptile, the bearded dragon (*Pogona vitticeps*). Eggs, from one clutch, were incubated at two different average temperatures within the normal range. After hatching the lizards were raised under the same environmental conditions. Novel object and novel environment tests were used to assess personality. Each test was repeated in both the short term and the long term. The results revealed that incubation temperature did impact upon ‘boldness’ but only in the short term and suggests that, rather than influencing personality, incubation temperature may have an effect on the development of behavioral of oviparous reptiles at different stages across ontogeny.

## Introduction

Human-induced environmental change, such as global warming, is increasingly affecting the habitats of animals worldwide. Often, the first response that an animal can make in the face of environmental change is behavioral (Tuomainen and Candolin 2011). Changes in behavior allow an organism to adapt to its environment in a rapid and efficient manner. Behavioral plasticity may allow species to combat the effects of human-induced climate change and allow them the time to develop the genetic diversity required in their new environment (Pigliucci 2001). Within a species, differences in personality could influence how individual animals respond to human-induced climate change with different personalities more successful under different conditions (Koolhaas et al. 1999; Minderman et al. 2009).

The term ‘personality’ describes differences in behavioral traits between individual animals of the same species that are consistent over time and across contexts (Carere and Eens 2005). Animal personality has been shown to be heritable (Brown et al. 2007) and subject to both natural and sexual selection (Smith and Blumstein 2008; Van Oers et al. 2005). Despite the underlying genetic component, the development of personality traits can also be heavily influenced by environmental factors, such as behavior of conspecifics (Frost et al. 2007) and predation pressure (Brown et al. 2005). Personality traits are measured along a continuum on a number of axes, e.g., boldness/shyness, proactive/reactive, explorative/less explorative (Sih and Del Giudice 2012; Carazo et al. 2014). Boldness, the willingness of an individual to take risks in a novel situation, has been investigated across various taxa, including fish (Frost et al. 2007); amphibians (Caelson and Langkilde 2013); reptiles (Carter et al. 2010); birds (Rokka et al. 2014); and mammals, (Carter et al. 2012). It has been linked to foraging success, mating success, and higher predation; it is thus considered to have far-reaching implications for an individual animal’s fitness (Biro and Stamps 2008; Wolf et al. 2007).

Despite the relative abundance of research into animal personality traits, the impact that early life experiences, particularly those which occur during embryonic development, have on animal post-hatching personality has received little attention. However, studies to date suggest that this is a promising avenue for research. Artificial manipulation of androgen levels in yolks of black-headed gull eggs (*Chroicocephalus ridibundus*) revealed a positive correlation between androgen levels and ‘aggression’ (Müller et al. 2009). Further, prenatal maternal effects, such as stress levels, have been shown to elevate glucocorticoid levels in both fish (McCormick 2006) and bird (Bertin et al. 2009) eggs and this is likely to impact upon offspring phenotype. (Rokka et al. 2014) found that the hormone composition of magpie (*Pica pica*) eggs, which is controlled by laying order, also impacts personality of the offspring. Taken together, current evidence suggests that prenatal effects do influence the personality of offspring.

Natural variation in egg incubation temperature impacts upon the phenotype of many oviparous reptiles in terms of sex, morphology, physiology, and behavior (for a comprehensive review see Deeming 2004; Booth 2006). The majority of behavioral work has investigated the impact of incubation temperature on either hunting or anti-predatory behavior (e.g., fleeing). Interestingly, the direction of the impact that this has upon behavior varies with species. For instance, cold-incubated hatchlings of the three-lined skink *Bassiana duperreyi* exhibited more anti-predatory behaviors than hot-incubated hatchlings (Flatt et al. 2001). By contrast, bearded dragons (*Pogona vitticeps*) incubated at hotter temperatures ran faster and were better at foraging than lizards incubated at colder temperatures (Siviter et al. in prep). Similarly, hatchling pine snakes (*Pituophis melanoleucus*) incubated at cool temperatures exhibited less anti-predatory responses and were less effective at foraging, when compared to hatchling snakes incubated at hotter temperatures (Burger 1991, 1998). Taken together, anti-predatory and exploratory behaviors do appear to be affected by incubation temperature, though the direction of the effect is not consistent across species.

Given that other behaviors are affected by natural variation in developmental environment, personality traits could, potentially, be also modulated by small differences in incubation environment. In reptiles, personality correlates with physiological measures known to be shaped by the incubation environment. For example, pigmentation can be modulated by the incubation environment in lizards and crocodilians (see Deeming 2004), and in a test that examined willingness to approach humans, darker melanin-based coloration correlates with boldness in Eastern Hermann’s tortoises (*Testudo hermanni boettgeri*; Mafli et al. 2011). Many species of reptile have temperature-dependent sex determination and sex-based personality differences have been observed in the Eastern water skink (*Eulamprus quoyii*), in which males were bolder than females (Carazo et al. 2014). Indeed, a recent study with bearded dragons (*Pogona vitticeps*) has revealed that incubating these lizards outside their normal range results in a shift from genotypic to environmental sex determination (Holleley et al. 2015). This shift has associated behavioral effects which appear to cause more male-like behavioral phenotypes in functionally female animals; this is likely to enhance fitness under certain conditions (Li et al. 2016). These findings indicate that incubation temperature may play an important role in shaping behavioral phenotypes, but it is difficult to disentangle the relative roles that incubation temperature and genetic differences played in the resultant differences in behavior. However, taken together, this further supports the idea that natural variation in the incubation environment may impact upon the personality of hatchlings.

Personality traits, such as boldness and exploration, are intrinsically linked to animal cognition. As the study of animal cognition investigates how animals perceive, store, and use information from the surrounding environment, cognition ultimately impacts upon most behaviors (Greggor et al. 2015). Recent research has started to directly investigate the link between cognition and personality and suggests that personality and cognitive performance within a species are linked (Sih and Del Giudice 2012; Carazo et al. 2014). Thus, the relationship between personality, particularly boldness, and cognitive style could be fundamental in understanding individual differences in cognitive performance (Sih and Del Giudice 2012; Guenther et al. 2014). Experimental work has revealed that performance on a spatial task was related to individual boldness levels in the Eastern water skink (*Eulamprus quoyii*; Carazo et al. 2014). Complementary to this, studies on the effect of incubation temperature on learning ability have shown that hot-incubated individuals were significantly better at learning tasks than cold-incubated animals in the three-lined skink (Amiel and Shine 2012; Amiel et al. 2014; Clark et al. 2014).

In order to understand individual variation in cognitive performance and to understand the influence of changing temperatures on oviparous reptiles, it is essential to investigate whether cognitive differences caused by incubation temperature may be the result of incubation temperature impacting relevant personality traits. In this experiment, we investigate the impact of egg incubation temperature on personality in lizards. This study looked at the effect of egg incubation temperature on the boldness of bearded dragons, an oviparous lizard found in central Australia. When incubated within the normal range, bearded dragons do not have temperature-dependent sex determination (Holleley et al. 2015) allowing us to discount sex as a factor affecting possible observed differences. We used both a novel object test and a novel environment test to investigate personality differences between animals incubated at different temperatures. The effect was examined in both the short term and the long term. Given the correlation between foraging success and boldness seen in other species, e.g., three-spined sticklebacks (*Gasterosteus aculeatus*; Ward et al. 2004) and barnacle geese (*Branta leucopsis*; Kurvers et al. 2009), we hypothesize that lizards incubated at a hot temperature would be bolder than lizards incubated at a cooler temperature.

## Methods

### Animals

This study was conducted using 13 bearded dragons hatched from the same clutch. The laying female only had access to one male. The eggs were randomly divided and incubated in multiple plastic boxes with vermiculite under two different heat conditions either 30 ± 3 °C, the ‘hot’ group, or 27 ± 3 °C, the ‘cold’ group. Humidity was kept high and constant for both conditions by regularly adding water to the vermiculite (see Booth 2004). Once the lizards had hatched, the environmental conditions, such as provision of food, housing, and room temperature, were kept the same. The lizards were originally kept in small vivariums measuring 20 × 30 × 30 cm; they were later moved to larger vivariums (145 × 48 × 60 cm) and were housed in groups. The lizards were maintained in the same room with an average temperature of 29 °C and were maintained on a 12:12 h light/dark cycle with lights turning on at 7 am. All animals were sexually mature at the initial time of testing and had previous experimental experience which measured various aspects of their foraging ability. They had not previously taken part in tests to assess personality. The lizards were handled daily, and they showed no behavioral signs of stress during handling. The ‘hot’ group contained 4 males and 3 females, while the ‘cold’ group contained 3 males and 3 females. The average mass of the ‘hot’ and the ‘cold’ groups did not differ significantly at the time of testing (average ± SE; hot: 220.8 ± 11.7 g; cold: 237.3 ± 17.7 g).

### Experiment 1: novel object experiment

The experimental arena was made of plastic-coated wood and measured 76.5 × 73 × 19 cm. Black meshing was placed over the top to ensure that the animals could not escape. The box was marked so that it was visually split into four quadrants (38.25 × 36.5 cm) one of which contained the novel item. Four objects, all novel to the lizards and all of which were a similar size (average ± SD; 13.62 ± 2.43 cm), were used in this experiment. The objects were a plastic toy police van, a china garden fairy, a pottery wellington boot, and a small plastic blue lamp. All four objects varied in color and were chosen so as not to be ecologically relevant (Greggor et al. 2015). All objects were sprayed with disinfectant to reduce potential olfactory cues, and the arena was cleaned between trials. The objects were always placed in the same quadrant in the arena, 54 cm from the starting position of the lizards (which remained the same throughout the experiment). The room was maintained at 28.5 ± 1 °C throughout testing. A digital camera (Sony HDR-CX22OE) was placed on a tripod above the arena so that the whole area was recorded during the experiment. The experimenter left the room for the duration of the trial.

#### Habituation

All animals were habituated to the testing arena prior to testing. During habituation, a lizard was placed into the arena for 15 min. After each 15-min period, a mealworm was placed on the opposite side of the enclosure. If the lizard ate the mealworm within 5 min on 3 consecutive days, then it was considered habituated. If the lizard failed to eat the mealworm within 5 min, the habituation procedure was repeated until the lizard met the criterion.

#### Testing

At the start of each trial, a lizard was placed on a marker on the diagonally opposite side of the arena to the object, facing toward it. In each Phase, lizards received two novel object trials (order of presentation counterbalanced across objects). Trials were separated by 4 or 5 days. Phase 1 trials were conducted when the animals were 10 months old, and the Phase 2 trials were a repeat of the experiment but using different novel objects 5 months after Phase 1.

### Experiment 2: novel environment experiment

This was identical to the previous experiment with some key changes. Instead of novel objects, different novel environments were used. Arena A measured 76.5 × 73 cm with pink patterned wrapping paper along the walls and sawdust on the floor. By contrast, arena B was rectangular (105 × 38 cm) and contained leopard print wall paper and a plastic bubble-wrap floor.

The lizards were not habituated to the experimental arena prior to the start of testing. Phase 1 trials were conducted when the animals were 12 months old. Phase 2 was conducted 3 months after Phase 1, and trials repeated the experiment using different novel environments: Arena C measured 77 × 38 cm with a laminated, patterned floor and a comic book wallpaper design. Arena D was 63 × 40 cm and contained a patterned wallpaper and the floor was covered with shredded paper.

### Data collection and statistical analysis

Solomon coder (© András Péter) was used to analyze the behavior of the lizards. All videos were coded by a coder who was blind to the experimental and incubation conditions. Twelve percent of the videos were second coded and a Spearman’s rank correlation revealed a strong positive correlation between observers (rho_23_ = 0.987, *p* < 0.001).

The first 5 min of each video was analyzed to allow comparison between the first and second exposure to the novel object. For the novel object experiment, the amount of time that each lizard spent in the quadrant surrounding the novel object was recorded. An animal was considered to be in the quadrant when any part of the lizard, with the exception of the tail, was on, or within, the quadrant line. When an animal was in the quadrant, it was considered as being in close proximity to the object. The time the lizard spent in locomotion was also recorded for each animal irrespective of location. It was considered to be in locomotion if it lifted a leg off the ground and the body position moved. A lizard was not considered to be moving if only its head moved. For the novel environment experiment, only locomotion was analyzed.

A general linear model with Gaussian errors and an identity link function (Minitab, ver. 17) was used to see if there was an effect of egg incubation ‘temperature’ (hot versus cold), and ‘Phase’ of the experiment (1 vs. 2), or any interaction between these fixed factors. Given the repeated measurement of the behavioral measures, individual lizards (‘subject’) were controlled in the model as a random factor nested within temperature. The analysis investigated the effects of these factors on the time spent in close proximity to the novel object, time spent moving in an arena with a novel object, or time spent moving around in a novel environment. Repeatability is defined as the proportion of phenotypic variation explained by differences between individuals, and so can provide a useful measure of behavioral consistency. We determined repeatability for each behavior in both the long term (Phase 1 to Phase 2) and short term (between trials in Phase 1 and Phase 2) using the method described by Lessels and Boag (1987), which allows the between- and within-individual components of variance to be estimated using analysis of variance. Possible values of repeatability range from zero to one.

## Results

### Experiment 1: novel object experiment

**Fig. 1:**
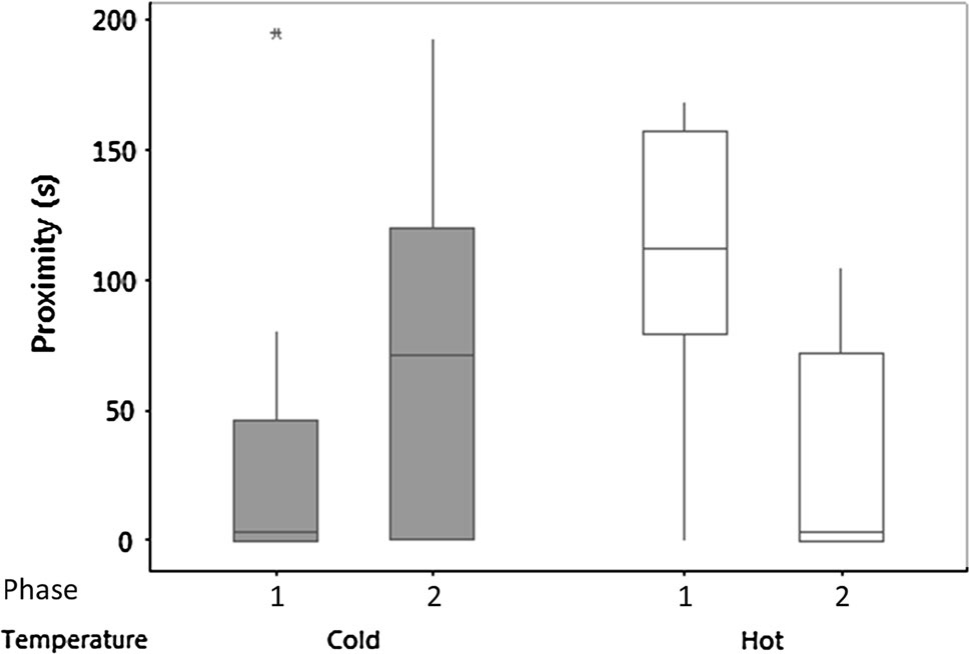
Novel object experiment: box and whisker plots of median time in seconds (*± sign* the interquartile range, *asterisk* outlier) spent in the quadrant containing the novel object for the ‘hot’ and ‘cold’ groups when the animals were first tested with two novel objects in Phase 1 and Phase 2

For the comparison between time spent in proximity to the novel object, neither temperature nor Phase as a fixed factor was significant but there was a highly significant interaction (Table 1). In Phase 1, lizards from the hot group spent more time in proximity to the object but this was reversed in Phase 2 (Fig. 1). Long-term repeatability was low, but repeatability in the short term was high in Phase 1 but low in Phase 2 (Table 2).

**Table 1:** Results of general linear model analysis to test the effect of temperature and phase as fixed factors, with subject nested within temperature as a random factor, on proximity to a novel object, locomotion when exposed to a novel object, and locomotion in a novel environment

	Proximity to novel object	Locomotion in arena with novel object	Locomotion in novel environment
	*F*_1,36_ (*P* value)	*F*_1,36_ (*P* value)	*F*_1,36_ (*P* value)
Temperature	0.81 (0.388)	0.37 (0.558)	1.07 (0.322)
Phase	1.93 (0.174	0.18 (0.678)	2.81 (0.103)
Temperature*phase	19.89 (<0.001)	0.21 (0.647)	1.49 (0.230)

**Table 2:** Short- and long-term behavioral repeatability

Repeatability (*r)*	Proximity to novel object	Locomotion in arena with novel object	Locomotion in novel environment
Short term (Phase 1)	0.491	0.185	0.598
Short term (Phase 2)	0.028	0.292	0.958
Long term	0.026	0.803	0.162

**Fig. 2:**
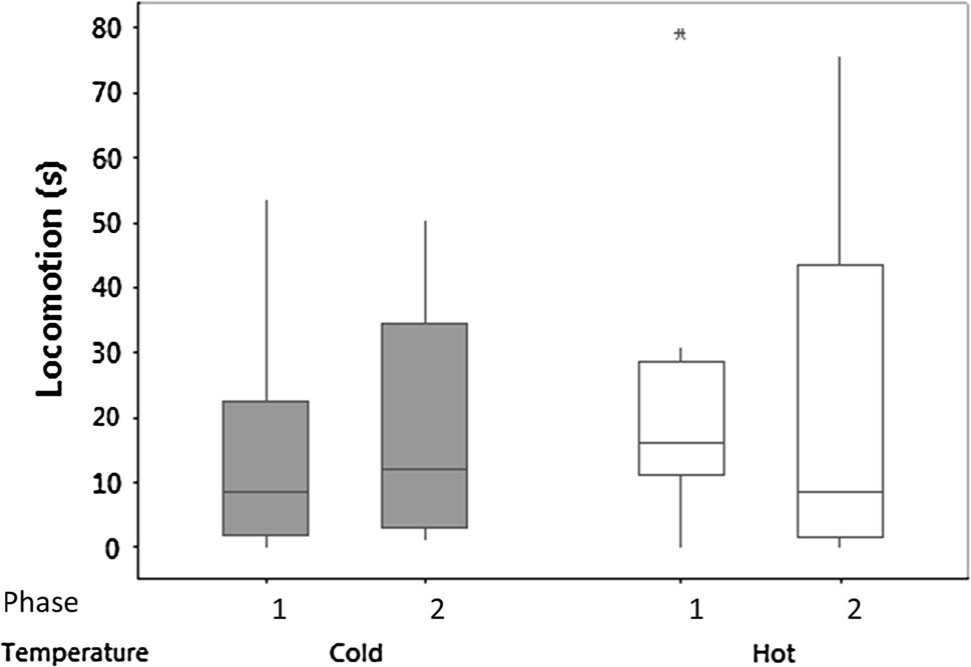
Novel object experiment: box and whisker plots of median time in seconds (*± sign* the interquartile range, *asterisk* outlier) spent in locomotion in the first novel object experiments tested in Phase 1 and 2 of the experiment

The time that the lizards spent in locomotion in the presence of a novel object was not significantly affected by incubation temperature, Phase, or their interaction (Fig. 2; Table 1). Repeatability in the long term was very high but was lower in the short term in both Phase 1 and Phase 2 (Table 2).

### Experiment 2: novel environment experiment

**Fig. 3:**
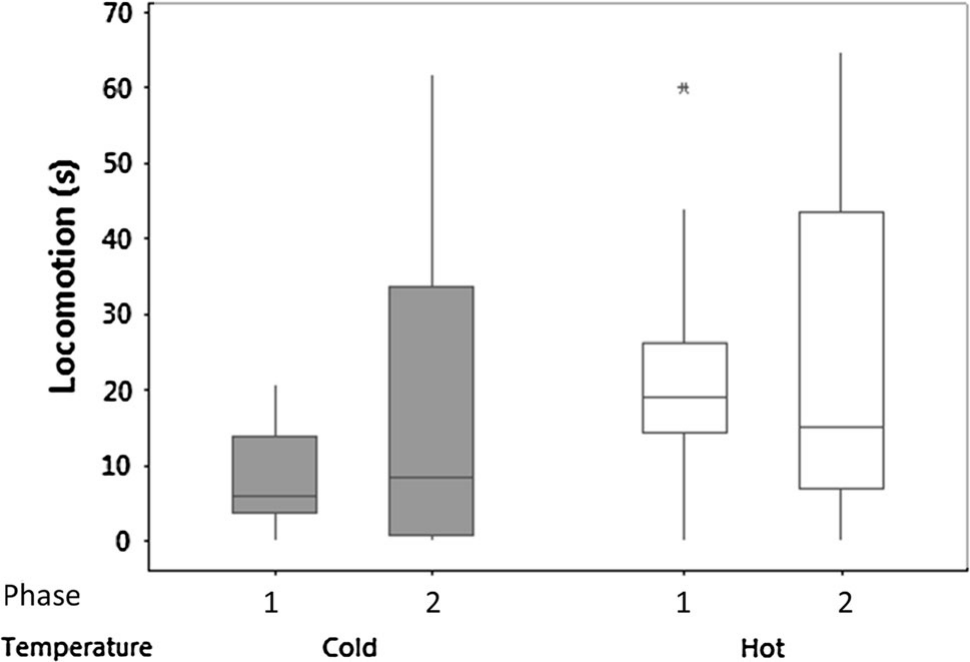
Novel environment experiment: box and whisker plots of median time in seconds (*± sign* the interquartile range, *asterisk* outlier) spent in locomotion for the ‘hot’ and ‘cold’ groups when the animals were tested with two novel environments in Phase 1 and 2 of the experiment

The time that the lizards spent in locomotion in a novel environment was not significantly affected by incubation temperature, Phase, or their interaction (Fig. 3; Table 1). In the short term, repeatability values were high for both Phases but were lower in the long term (Table 2).

## Discussion

Our results represent the first data to show that natural variation in environmental factors during embryonic development, such as incubation temperature, may impact upon the development of behavioral traits during ontogeny. For behavioral traits, such as boldness, to be defined as personality, they need to be repeatable across time and context (Carere and Eens 2005). We investigated this by examining the impact of incubation temperature on the behavior of lizards toward a novel object and in a novel environment. Each was assessed over two different time periods short term (4 or 5 days) and long term (at least 3 months). We saw no long-term impact of incubation temperature on behavior; however, incubation temperature did impact upon behavior in the short term. In the novel object experiment, the ‘hot’ group initially spent more time in close proximity to the novel object than the ‘cold’ group; however, the opposite was true when tested in the later phase of the experiment. There was no effect of incubation temperature on the amount of locomotion in either the novel object experiment or the novel environment experiment. The results suggest that, rather than impacting upon personality, egg incubation temperature influenced the development of behavioral traits.

Interestingly, the majority of experiments testing animal personality look at repeatability of behavioral traits over short periods of time (e.g., Frost et al. 2007; Short and Petren 2008). The findings of our study highlight the importance of re-testing animals over a longer period. Had this experiment failed to test the long-term repeatability, a different interpretation would have been placed on the results. However, one important consideration in interpretation of these data is the limited sample size used in this experiment. Some of the variations observed in the data could be attributed to this, and replication with additional animals is an important next step. Additionally, only one clutch was used in this experiment, and as such it is possible that the results are not generalizable to the population. We accept that the results are based on a small sample size from one clutch of eggs, but we feel that they are sufficiently interesting to stimulate further research.

Egg incubation temperature has been shown to affect the development of oviparous reptiles in terms of growth, physiology, and behavior (Deeming 2004). For example, egg incubation temperature affects the growth rates of bearded dragons at different stages in ontogeny (Siviter et al. in prep). As hatchlings, the lizards incubated at hotter temperatures were heavier than animals incubated at colder temperatures. However, as the animals developed, those incubated at colder temperatures grew heavier significantly faster (Siviter et al. in prep). Surprisingly, developmental changes in the behavior of oviparous reptiles as a result of incubation history have rarely been examined. The majority of previous studies only tested the behavior of hatchlings (see Deeming 2004; Booth 2006), and as a result, the long-term effects of incubation temperature on behavior are largely unknown. A recent study has revealed that incubating bearded dragons outside their normal range results in a shift from genotypic to environmental sex determination (Holleley et al. 2015) and that this shift has associated behavioral effects (Li et al. 2016). However, in this study it is unclear whether the differences in behavior are the result of incubation temperature or genetic differences. By comparing the behavioral responses of bearded dragons in both the short term and the long term, our results provide the first insight into the impact that incubation temperature alone has on personality and the development of and behavioral traits.

Personality traits, such as boldness and activity, have been shown to be linked with food acquisition in a range of different species (for a review see Biro and Stamps 2008). The results of the present experiment suggest that incubation temperature may impact upon the development of behavioral traits of bearded dragons such that they differ at different stages of their ontogeny. It is therefore likely that they will strongly influence their success. A positive correlation between foraging intake and boldness or exploration has been observed in a range of animals (e.g., in the great tit (*Parus major*; Naguib et al. 2010) and in the common sole (*Solea*; Mas-Muñoz et al. 2011). In addition to foraging success, boldness has been positively correlated with basking time in the Namibian rock agama (*Agama planiceps*; Carter et al. 2010) and reproductive success in the Southern water skink (*Eulamprus heatwolei;* Keogh et al. 2012). However, when resources are plentiful Namibian rock agama that are less bold may have an advantage over active lizards, which may have a higher likelihood of predation (Carter et al. 2010) and will potentially use more energy. Cooler egg incubation temperatures can produce heavier and larger lizards (see Deeming 2004) suggesting that lizards incubated at these temperatures will be able to dominate resources (Tokarz 1985) and retain heat for longer.

The mechanisms that cause temperature-dependent differences in the phenotypes of oviparous reptiles are still not fully understood (Valenzuela 2004; Deeming 2004). One, as yet unexplored suggestion, is that incubation temperatures differentially impact upon the development and physiology of the hypothalamus, which has a crucial role in regulation of a variety of bodily functions in vertebrates including growth and thermoregulatory behavior (Deeming and Ferguson 1989). Irrespective of the mechanism, it is possible that the relationship between egg incubation temperature and the phenotype of oviparous species of reptiles is adaptive; hatchlings from a clutch of eggs exposed to a range of temperatures within the nest environment will be suited to a wide variety of different environments (Deeming and Ferguson 1989). Moreover, the environment in which the lizards are incubated is reflective of the environment that they are emerging into. Therefore, a warmer environment may produce animals that are better adapted to survival in that temperature profile. As such, it has been suggested that the sensitivity of oviparous reptiles to environmental factors may make animals more flexible in dealing with changing environments and could be a valuable defense mechanism for tackling human-induced climate change (Holleley et al. 2015). Such effects may be mediated through the impact that incubation temperatures have on the phenotypes of hatchlings and older animals. Behavioral responses to ontogenetic variation in lizards need more investigation in a wide range of species to help us understand not only reptilian cognition but also the possible effects of anthropogenic climate changes.
